# Closed-Form Results for Prior Constraints in Sum-Product Networks

**DOI:** 10.3389/frai.2021.644062

**Published:** 2021-04-08

**Authors:** Ioannis Papantonis, Vaishak Belle

**Affiliations:** ^1^School of Informatics, University of Edinburgh, Edinburgh, United Kingdom; ^2^The Alan Turing Institute, London, United Kingdom

**Keywords:** sum-product networks, constraints, tractable models, optimization, machine learning

## Abstract

Incorporating constraints is a major concern in probabilistic machine learning. A wide variety of problems require predictions to be integrated with reasoning about constraints, from modeling routes on maps to approving loan predictions. In the former, we may require the prediction model to respect the presence of physical paths between the nodes on the map, and in the latter, we may require that the prediction model respect fairness constraints that ensure that outcomes are not subject to bias. Broadly speaking, constraints may be probabilistic, logical or causal, but the overarching challenge is to determine if and how a model can be learnt that handles a declared constraint. To the best of our knowledge, treating this in a general way is largely an open problem. In this paper, we investigate how the learning of sum-product networks, a newly introduced and increasingly popular class of tractable probabilistic models, is possible with declared constraints. We obtain correctness results about the training of these models, by establishing a relationship between probabilistic constraints and the model's parameters.

## 1. Introduction

Incorporating constraints is a major concern in data mining and probabilistic machine learning (Raedt et al., 2010; Kisa et al., 2014; Friedman and Van den Broeck, 2019). A wide variety of problems require the prediction to be integrated with reasoning about various forms of constraints, ranging from constraining the support of a distribution, such as when modeling routes on maps (Shen et al., 2018; Xu et al., 2018), to enforcing certain independence relationships, such as when approving loan predictions (Mahoney and Mohen, 2007). That is, when modeling routes, we may require the prediction model to respect the presence of physical paths between nodes on the map, in the sense of assigning zero probability to impossible or infeasible paths. Analogously, when approving loans, we may have conditional constraints for eliminating bias, e.g., the prediction should be independent of the applicant's ethnicity or gender.

Broadly, background information may come in different forms, including independency (Zemel et al., 2013; Zafar et al., 2015) constraints and logical formulas (Kisa et al., 2014; Xu et al., 2018), but of course the challenge is if and how we are able to provide (or learn) a model that is able to handle the declared constraint. To the best of our knowledge, this is largely an open problem, at least in the sense of providing a general solution to a certain class of probabilistic models.

In addition to incorporating prior knowledge as constraints for training a probabilistic model, a second and equally significant way to utilize constraints is in order to enforce a set of properties on the resulting models. For example, historic data on college admissions exhibit a clear bias based on gender or race (Leonard and Jiang, 1999; Silverstein, 2000). More generally, there is an abundance of data that reflect historical or cultural biases, prompting the rapid development of the area of fair machine learning (Zafar et al., 2015; Hardt et al., 2016). Roughly, the idea is to place a constraint (e.g., a formalization that captures, for example, demographic parity Zafar et al., 2015 or equality of opportunity Hardt et al., 2016) on the predictions of the resulting model so that biased behavior is not exhibited.

In this paper, we investigate the definability of constraints while training/learning a probabilistic model. Note however that performing inference on probabilistic models is a computationally intractable problem (Bacchus et al., 2009), requiring additional, often computationally intensive, subroutines in order to approximate inference. This has given rise to tractable probabilistic models (TPMs) (Poon and Domingos, 2011; Kisa et al., 2014) where conditional or marginal distributions can be computed in time linear in the size of the model. Although initially limited to low tree-width models (Bach and Jordan, 2002), recent tractable models, such as sum product networks (SPNs) (Poon and Domingos, 2011; Gens and Domingos, 2013) and probabilistic sentential decision diagrams (PSDDs) (Kisa et al., 2014; Liang et al., 2017) are derived from arithmetic circuits (ACs) and knowledge compilation approaches, more generally (Darwiche, 2002; Choi and Darwiche, 2017), which exploit efficient function representations and also capture high tree-width models. These models can also be learnt from data (Gens and Domingos, 2013; Liang et al., 2017) which leverage the efficiency of inference. Consider that in classical structure learning approaches for graphical models, once learned, inference would have to be approximated, owing to its intractability. In that regard, such models offer a robust and tractable framework for learning and inferring from data. Owing to these properties and their increasing popularity for a wide range of applications (Poon and Domingos, 2011; Choi et al., 2015; Liang and Van den Broeck, 2019) and several extensions have been explored as well (Molina et al., 2018; Shen et al., 2018). We focus on SPNs, over descrete variables, in this work, but our approach could be extended to other TPMs. We aim at targeting PSDDs in future work, since they already allow for incorporating logical constraints, so an extension to handling logical along with probabilistic and causal constraints could be very significant.

We are organized as follows: we first review the recent advances in constrained machine learning. Then we briefly review SPNs, and some preliminaries on constrained optimization. We then turn to our main results. Finally, we conclude with discussions.

## 2. Related Work and Context

During the last years, there have been ongoing attempts to address the problem of incorporating constraints during training or in prediction. For example, Xu et al. (2018) examine the problem of imposing certain structure in the outcome of a classification algorithm. They approach this by adding an additional term in the objective function, one accounting for the probability of a state satisfying the given constraint. Marquez Neila et al. (2017) consider the case of training a neural network under some constraints. They create two variants of this problem, one where results from optimization theory are utilized in order to efficiently solve the problem, under hard constraints, as well as a relaxation of this problem, with soft constraints (Gill et al., 1981; Fletcher, 1987), where terms corresponding to the constraints are added into the objective function.

Alternative ways to utilize prior knowledge have been proposed as well, such as Stewart and Ermon (2017). In this work, the authors propose a framework for the semi-supervised training of neural networks. The key insight is that pre-existing knowledge can be used to create a regularizer, prompting the network to satisfy this information.

Data mining is an other field that utilizes constraints. For example, Raedt et al. (2010) attempt to develop a structured way to apply constrained programming techniques in pattern mining or rule discovery.

Introducing constraints as a way to control a model's complexity has been explored as well. Friedman and Van den Broeck (2019) consider an approach where they constrain the expected value of a quantity, modeled using open-world probabilistic databases (Ceylan et al., 2016). By doing that, they go on to show how this constraint strengthens the semantics of such databases.

Incorporating constraints has also be explored in the context of learning the structure of Bayesian models. For example, in Chen et al. (2016), the authors investigate ways to impose ancestral relationships between nodes. It is worth noting that, conceptually, this approach could also be useful in enforcing probabilistic constraints, since the graph's topology is sufficient to encode them. However, the amount of ancestral constraints that have to be considered, renders a straightforward application of this methodology infeasible for problems with relatively high dimensionality. In our approach, we do not implement independence through manipulating the paths between nodes, but by forcing the parameters to take on values in such a way that guarantees independence between variables.

Another line of research, can be found in Dechter et al. (1991), where the authors consider ways to uncover feasible solutions of problems with temporal constraints. Furthermore, in Dechter (1999), a framework is presented, dealing, among others, with the satisfiability of problems, under more general constraints. We would just like to note that our work proposes a way to design models with background probabilistic knowledge, while works like the above, explore whether a constraint problem has a solution.

Our contribution lies in introducing an approach for training generative models under probabilistic constraints. We borrow concepts from optimization theory and develop a paradigm related to Marquez Neila et al. (2017). A key difference is that their approach, although similar in spirit, takes into account constraints that are expressed in terms of the model's outcomes. Thus, they correspond to functional relationships that the output variables should respect, so, consequently, they are not of a probabilistic nature. In contrast, our approach provides a way for incorporating probabilistic constraints across all variables. Indeed, in the following sections, we will provide insights about the link between these constraints and the system of equations they induce.

In our proposed framework we suggest to utilize tractable probabilistic models (Poon and Domingos, 2011; Kisa et al., 2014), where conditional or marginal distributions can be computed in time linear in the size of the model, so we can efficiently answer the conditional or marginal queries that come up when incorporating constraints. Specifically, we will base our presentation on sum-product networks (SPNs) (Poon and Domingos, 2011). SPNs are instances of arithmetic circuits (ACs) (Choi and Darwiche, 2017) that compactly represent the network polynomial (Darwiche, 2003) of a Bayesian network (BN).

In this paper we explore the following: can SPNs be used in order to train generative models subject to probabilistic and causal constraints? We demonstrate how to incorporate various types of probabilistic relationships into the model, specifically targeting hard and soft constraints. To do so, we show how constraining the model's parameters to satisfy a system of equations, guarantees that the resulting model satisfies the desired relationships.

## 3. Background

In this section we will briefly review SPNs, some causality related concepts, as well as some optimization approaches.

### 3.1. SPNs

SPNs are rooted directed graphical models that provide for an efficient way of representing the network polynomial (Darwiche, 2003) of a BN (Poon and Domingos, 2011), as a multilinear function $\underset{\text{x}}{\sum }f\left(\text{x}\right)\prod_{n=1}^{N}{𝟙}_{x_{n}}$. Here *f*(·) is the (possibly unormalized) probability distribution of the BN, **x** is a vector containing all the variables of the model, i.e., *x*_1_, ⋯ , *x*_*N*_, the summation is over all possible states, and 𝟙_*x*_*n*__ is the indicator function. In this section, we are going to present the class of binary SPNs, but it is immediate to extend the definitions to discrete variables with an arbitrary number of values. Taking this into account, the network polynomial contains 2^*N*^ terms, in its simplest form, but there is a wide array of problems, where it is possible to obtain a factorized representation, that is not exponential in the number of the model's variables. This is exactly the idea behind SPNs, discovering a compact factorization of the network polynomial, enabling inference to be performed in a highly efficient manner.

An SPN S over Boolean variables *x*_1_, ⋯ , *x*_*N*_ has leaves corresponding to indicators 𝟙_*x*_1__, ⋯ , 𝟙_*x*_*n*__ and ${𝟙}_{{\bar{x}}_{1}},\cdots \;,{𝟙}_{{\bar{x}}_{n}}$ and whose internal nodes are sums and products. Any edge exiting a sum node has a non-negative weight assigned to it. The value of a product node is the product of its children, while the value of a sum node is a weighted sum of its children, $\underset{u_{j}\in Ch\left(u_{i}\right)}{\sum }w_{ij}S_{j}\left(\text{x}\right)$, where *Ch*(*u*_*i*_) is the set containing the children of node *u*_*i*_, and S_*j*_ is the sub-SPN rooted at node *u*_*j*_. We can define an SPN, as follows:

- Any tractable univariate distribution is an SPN (this corresponds to the base case).
- The product of two SPNs with disjoint set of variables is also an SPN (this can be seen as the factorization of independent distributions).
- The weighted sum of two SPNs with the same set of variables is an SPN, too (denoting a mixture of distributions).

There has been a number of algorithms developed for training SPNs, such as in Poon and Domingos (2011), where SPNs were firstly introduced. In this work, a dense SPN is initialized, followed by iteratively updating its parameters, utilizing gradient information, until a stopping criterion is met. Once the training is completed, all edges having zero weights are pruned, and all the nodes that become unreachable due to this are deleted, uncovering the final structure of the SPN. Another approach can be found in Gens and Domingos (2013), where the algorithm starts with a single node representing the entire dataset, and recursively adds product and sum nodes that divide the dataset into smaller datasets until convergence. Product nodes are created using group-wise independence tests, while sum nodes are created performing clustering on the row instances. The weights associated with sum nodes are learned as the proportion of instances assigned to a cluster.

**Figure 1:**
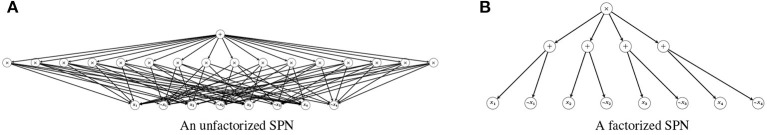
A naive implementation of a SPN vs. a SPN that takes into account the independence among the variables. **(A)** An unfactorized SPN. **(B)** A factorized SPN.

The non-factorized representation of a network polynomial, $\underset{\text{x}}{\sum }f\left(\text{x}\right)\prod_{n=1}^{N}{𝟙}_{x_{n}}$, is also called the *canonical* polynomial, and it is unique, in the sense that two SPNs with the same canonical polynomial are identical (Darwiche, 2003). Having said that, these representations are mostly theoretical tools, since they require exponential space, rendering them impractical for most applications. This is why structure learning algorithms have focused on obtaining factorized representations of the underlying canonical polynomial, in order to reduce the space complexity. However, there can be multiple ways to factor a canonical polynomial, meaning that the compactness of the resulting factorization has a great impact on the performance of the resulting SPN. Figure 1 contrasts a SPN that directly encodes the canonical polynomial of the distribution of four independent variables to one that takes the independence assumption into account. Both SPNs have the same output for any given assignment, but the SPN in (1*b*) is clearly more compact.

### 3.2. Causality

Causal inference is an approach where, apart from probabilistic information, extra information about the mechanism governing the variables' interactions are encoded into the model. This allows reasoning about more complex queries, such as interventions and counterfactuals (Pearl, 2009b). These queries extend standard probabilistic reasoning (marginalization and conditioning) with the ability to infer what happens if a variable is forced to attain a value, by an external intervention, or what would happen had a variable obtained a different value from the one it obtained in the actual world.

The usual setting is to represent the set of probabilistic dependencies through a BN, but on top of that encode the specific mechanism that determines the value of each variable, too. In this sense, it is more general than just having a BN, since we not only possess a distribution over the variables, but also a set of equations.

An interesting remark is that, although the structural equations connecting the variables are essential for the specification of the model, it turns out that specifying the variables' distribution, alone, is sufficient for answering interventional queries (Pearl, 2009a). In our approach we are going to utilize the following formula to compute the effect of intervening on a variable, *A*, on the rest of the model's variables, **X**_−*A*_ (Pearl, 2009b):

$$\begin{array}{l} \mathrm{Pr}\left(X_{-A}|do\left(A=\alpha \right)\right)=\frac{\mathrm{Pr}\left(X_{-A},A=\alpha \right)}{\mathrm{Pr}\left(A=\alpha |pa_{A}\right)} \end{array}$$

where *pa*_*A*_ denotes the set of A's parents.

### 3.3. Optimization

**Figure 2:**
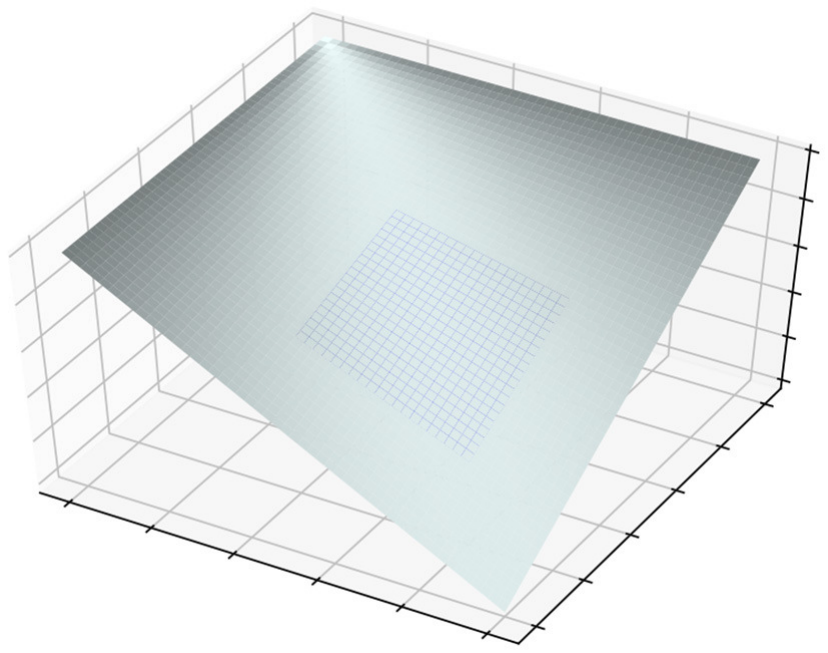
An example of optimizing a function, while constraining the solution to lie on the shaded part.

Constrained optimization is a discipline concerned with developing techniques allowing for optimizing functions under a set of constraints. For example, Figure 2 depicts the problem of minimizing a function, while requiring the solution to belong to the shaded area. One of the most common ways to address that, is to transform the objective function, so it takes the constraints into account. The problem of interest is to maximize the likelihood of a model (with a vector of parameters **w**), *L*(**w**) under constraints *C*_*i*_(**w**) = 0, 1 ≤ *i* ≤ *N*, so:

$$\begin{array}{l} max_{w}L\left(w\right),\;s.t.C_{1}\left(w\right)=0,\cdots \;,C_{N}\left(w\right)=0 \end{array}$$

The transformed objective function, Λ, introduces a number of auxiliary variables, as many as the constraints, λ_1_, ⋯ , λ_*N*_, and takes the following form $\Lambda \left(\text{w},\lambda_{1},\cdots \;,\lambda_{N}\right)=L\left(\text{w}\right)+\overset{N}{\underset{n=1}{\sum }}\lambda_{n}C_{n}\left(\text{w}\right)$. It can be shown that all of the solutions of the original problem correspond to stationary points of the new objective function (Protter and Morrey, 1985).

There are various numerical methods to solve this problem, such as projected gradient descent, where an initial vector **w**^(0)^ is updated incrementally, and then gets projected onto the surface defined by the constraints, until it converges to a solution of the problem. Furthermore, in cases where the objective function is in a special form, such as a quadratic polynomial, other approaches might be more efficient. See Marquez Neila et al. (2017) for a more extensive discussion on the subject.

Alternative ways to address constraint optimization problems include recent advances, such as Cotter et al. (2019a,b), where the optimization objective is formulated as a game between two players. Approaches like these can be readily incorporated within our framework, since we are going to make use of only differentiable constraints, as we will see in what follows.

Optimization problems like the above require all of the feasible solutions to satisfy the constraints. These constraints are referred to as *hard*. Alternative formulations of the problem could yield feasible solutions not satisfying the constraints. These constraints are called *soft*, because instead of demanding the solutions to adhere to them, we introduce a penalty term in the objective function, for each time they get violated. For example, if all of the *C*_*i*_(**w**) = 0, 1 ≤ *i* ≤ *N* were treated as soft constraints, then after setting λ_1_, ⋯ , λ_*N*_ to some value reflecting the cost of violating the corresponding constraint, the soft version of the problem would be to maximize the function $L\left(\text{w}\right)+\overset{N}{\underset{n=1}{\sum }}\lambda_{n}C_{n}\left(\text{w}\right)$, so each time some *C*_*i*_ is not equal to zero, it induces a penalty. In this case, all λ_*i*_ are treated as hyperparameters, so they are specified before the optimization takes place. Furthermore, now we are interested in the maxima of this function, as opposed to the case of hard constraints, where we were interested in the stationary points of the transformed function.

## 4. Main Results

The majority of contemporary machine learning models rely on maximum likelihood (ML) estimation for setting the values of their parameters. The approaches we discussed earlier transform the optimization objective, enhancing the resulting model with additional properties. One limitation, in such a setting, is that the constraints are expressed in terms of the parameters, directly (Marquez Neila et al., 2017). This is useful in situations where we require some parameters to be equal to each other, or their difference to exceed some threshold. However, in most models it is not clear how probabilistic relationships can be expressed in term of the parameters, making it difficult to utilize the existing approaches in order to achieve our goal.

Our approach is motivated from such formulations, but appeals on the following idea: identifying a class of models where it is feasible to uncover a correspondence between parameters and probabilities would enable the *use of constrained optimization approaches, in order to equip the model with additional properties*. The modeler would provide the constraints in terms of the variables modeling the domain, i.e., the random variable in a generative model; arguably, this is a natural and intuitive way to express domain knowledge.

Most probabilistic constraints are expressed as an equality between probabilities. For example, if we want to incorporate the assumption that “*A* is independent of *B*,” we have to ensure that the equality Pr(*A, B*) = Pr(*A*) Pr(*B*) holds in the trained model.

### 4.1. Conditional Constraints

We will start with presenting the case of constraining the likelihood so it enforces equality between various conditional distributions. Formally, assume a variable *Y*, a variable *A*, whose values we would like to condition on, and a set of variables, *X*. We are interested in modeling the joint distribution of these variables, but we would also like to incorporate some background knowledge into the model, specifically we would like it to satisfy the condition Pr(*Y*|*A* = α, **X**) = Pr(*Y*|*A* = α′, **X**), where we assume that *A* is a binary variable, in order to make the presentation easier to follow. In this equation we do not explicitly specify the values of the variables in *X*, rather we want the condition to hold regardless of their specific instantiation. We could also be interested in constraints of the form Pr(*Y*|*A* = α) = Pr(*Y*|*A* = α′), where in this case we do not condition on *X*. Constraints similar to this appear in the fair AI literature (Zemel et al., 2013; Zafar et al., 2015; Grgić-Hlača et al., 2016; Hardt et al., 2016), where the objective is to eliminate bias, such as racial discrimination, from predictive models, by enforcing an appropriate set of conditions. For example, *Y* could represent the outcome of a loan application, while *A* could represent the applicant's ethnicity. The goal of a conditional constraint, then, would be to make sure that the probability of granting a loan application is the same for all ethnic groups. We should also note that such properties cannot be imposed at inference time, since even the marginal distribution of *Y* might have been affected by information regarding the protected attribute, which leaked during training. This situation could arise under a variety of circumstances, such as when training a model using an imbalanced dataset (Sapiezynski and Valentin Kassarnig, 2017; Mehrabi et al., 2019), in which case conditional constraints can be utilized to impede such information leakage from happening.

An additional remark about the flexibility of expressing constraints in this form can be seen when considering context-specific properties (Zhang and Poole, 1999). In the above formulation we left the values of *X* unspecified, but there might be cases where it is known that some properties hold only when some of the remaining variables acquire specific values. To take such information into account we should just adapt the constraint so some or all of the variables in *X* are set to their corresponding values, for example, such a constraint could look like Pr(*Y*|*A* = α, **X** = **x**) = Pr(*Y*|*A* = α′, **X** = **x**).

As we have stated above, we are going to use SPNs to model the data, due to their provable tractability and applicability in a wide range of problems and the fact that a clean connection between probabilistic queries and the model's parameters can be established. This is crucial for our approach, since, in general, it is not clear how to achieve this connection. However, the polynomial representation of SPNs allow us to uncover it and use it to train such a model under a set of probabilistic constraints. The only essential requirement for our results is that there should be no a priori parameter tying assumptions, in the SPN, since that would affect the degree of the obtained system of equations. Arguably, this is not a major restriction, since no SPN learning algorithm makes this assumption. To our knowledge, the only case where parameter tying is used in SPNs, is to encode head-to-head structures, when transforming a BN into a SPN (Peharz et al., 2015). However, in practice SPNs are learnt from data, not by transforming an underlying BN, so this kind of situation does not usually arise. Nevertheless, it should still be possible to establish a connection between constraints and the SPN's parameters, in this case as well, although we are not going to consider this case in this work.

The following results establishes the relationship between probabilistic constraints and the parameters of an SPN, **w** (An analogous statement applies to the other variants discussed above).

**Theorem 1**. *Let* S *be an SPN representing the joint distribution of variables X*_1_, ⋯ , *X*_*n*_. *Let X*_*i*_, *X*_*j*_ *be two binary variables, then the constraint* Pr(*X*_*i*_|*X*_*j*_ = 0) = Pr(*X*_*i*_|*X*_*j*_ = 1) *is equivalent to a multivariate linear system of two equations on the SPN's parameters*.

*Proof*. Let $S\left(\text{x}\right)=\underset{\text{x}}{\sum }f\left(\text{x}\right)\prod_{n=1}^{N}{𝟙}_{x_{n}}$ be the network polynomial of an SPN. The equality Pr(*X*_*i*_|*X*_*j*_ = 1) = Pr(*X*_*i*_|*X*_*j*_ = 0) can be rewritten as follow:

(1) $$\begin{array}{l} \mathrm{Pr}\left(X_{i}|X_{j}=1\right)=\mathrm{Pr}\left(X_{i}|X_{j}=0\right)\Rightarrow \frac{\mathrm{Pr}\left(X_{i},X_{j}=1\right)}{\mathrm{Pr}\left(X_{j}=1\right)} \end{array}$$

$$\begin{array}{l} =\frac{\mathrm{Pr}\left(X_{i},X_{j}=0\right)}{\mathrm{Pr}\left(X_{j}=0\right)}\Rightarrow \mathrm{Pr}\left(X_{i},X_{j}=1\right)\cdot \mathrm{Pr}\left(X_{j}=0\right)\\ =\mathrm{Pr}\left(X_{i},X_{j}=0\right)\cdot \mathrm{Pr}\left(X_{j}=1\right) \end{array}$$

Next, we express the above probabilities in terms of S (where *X* corresponds to the assignment *X* = 1, and ¬*X* to *X* = 0):

$$\begin{array}{l} \mathrm{Pr}\left(X_{i},X_{j}=1\right)=\underset{x:x_{i},x_{j}}{\sum }f\left(x\right){𝟙}_{x_{i}}+\underset{x:\neg x_{i},x_{j}}{\sum }f\left(x\right){𝟙}_{\neg x_{i}}\\ \mathrm{Pr}\left(X_{i},X_{j}=0\right)=\underset{x:x_{i},\neg x_{j}}{\sum }f\left(x\right){𝟙}_{x_{i}}+\underset{x:\neg x_{i},\neg x_{j}}{\sum }f\left(x\right){𝟙}_{\neg x_{i}}\\ \mathrm{Pr}\left(X_{j}=1\right)=\underset{x:x_{j}}{\sum }f\left(x\right)\\ \mathrm{Pr}\left(X_{j}=0\right)=\underset{x:\neg x_{j}}{\sum }f\left(x\right) \end{array}$$

We now substitute these equations to (2) to get that:

$$\begin{array}{l} \underset{x:\neg x_{j}}{\sum }f\left(x\right)\cdot \underset{x:x_{i},x_{j}}{\sum }f\left(x\right){𝟙}_{x_{i}}+\underset{x:\neg x_{j}}{\sum }f\left(x\right)\cdot \underset{x:\neg x_{i},x_{j}}{\sum }f\left(x\right){𝟙}_{\neg x_{i}}=\\ \underset{x:x_{j}}{\sum }f\left(x\right)\cdot \underset{x:x_{i},\neg x_{j}}{\sum }f\left(x\right){𝟙}_{x_{i}}+\underset{x:x_{j}}{\sum }f\left(x\right)\cdot \underset{x:\neg x_{i},\neg x_{j}}{\sum }f\left(x\right){𝟙}_{\neg x_{i}} \end{array}$$

This is an equality between polynomials, meaning that the coefficients must be equal, so:

$$\begin{array}{l} \underset{x:\neg x_{j}}{\sum }f\left(x\right)\cdot \underset{x:x_{i},x_{j}}{\sum }f\left(x\right)=\underset{x:x_{j}}{\sum }f\left(x\right)\cdot \underset{x:x_{i},\neg x_{j}}{\sum }f\left(x\right)\\ \underset{x:\neg x_{j}}{\sum }f\left(x\right)\cdot \underset{x:\neg x_{i},x_{j}}{\sum }f\left(x\right)=\underset{x:x_{j}}{\sum }f\left(x\right)\cdot \underset{x:\neg x_{i},\neg x_{j}}{\sum }f\left(x\right) \end{array}$$

These constraints are expressed in terms of the model's parameters and they are multivariate linear polynomials, since in each equation there are two products, so if we look, for example, at the ones in the first equation, $\underset{\text{x}:x_{i},x_{j}}{\sum }f\left(\text{x}\right)\cdot \underset{\text{x}:\neg x_{j}}{\sum }f\left(\text{x}\right)$ and $\underset{\text{x}:x_{i},\neg x_{j}}{\sum }f\left(\text{x}\right)\cdot \underset{\text{x}:x_{j}}{\sum }f\left(\text{x}\right)$, the terms that appear in one factor don't appear on the other one, since the summation is performed over disjoint sets.

At this point, we should note that although the above result is stated for binary SPNs, to make the flow of the proof easier to follow, it holds for discrete SPNs, in general. For example, if *X*_*i*_ ∈ {0, 1} and *X*_*j*_ ∈ {0, 1, ⋯ , *k* − 1}, then the desired constraint takes the from Pr(*X*_*i*_|*X*_*j*_ = 0) = Pr(*X*_*i*_|*X*_*j*_ = 1) = ⋯ = Pr(*X*_*i*_|*X*_*j*_ = *k* − 1). These multiple equalities are equivalent to the following system of *k* − 1 equations: Pr(*X*_*i*_|*X*_*j*_ = 0) = Pr(*X*_*i*_|*X*_*j*_ = 1), Pr(*X*_*i*_|*X*_*j*_ = 1) = Pr(*X*_*i*_|*X*_*j*_ = 2), …, Pr(*X*_*i*_|*X*_*j*_ = *k* − 2) = Pr(*X*_*i*_|*X*_*j*_ = *k* − 1). The main observation here, is that each equation involves *X*_*i*_ and only two states of *X*_*j*_, so (a slight modification of) Theorem 1 applies to each equation, meaning that each of them induces 2 (multivariate linear) equations, so the overall number of equations in the system is 2(*k* − 1). The same reasoning can be extended to the general case, where *X*_*i*_ ∈ {0, 1, ⋯ , *m* − 1} and *X*_*j*_ ∈ {0, 1, ⋯ , *k* − 1}, resulting into a system of *m*(*k* − 1) equations, indicating that the number of equations scale linearly with respect to the product of the ranges of both variables.

### 4.2. Interventional Constraints

A more complex class of distributions, used extensively in causal modeling (Pearl, 2009a), are interventional ones. They represent the probability of a variable after an external intervention on another variable. It is not always possible to estimate them using observational distributions, but when assuming that all of the model's variables are observed, then it is possible to express the interventional distribution in terms of the observational one (Pearl, 2009b). For the rest of this section we will make the closed-world assumption, meaning that there are no unobserved confounders between the variables. Incidentally, causal modeling concepts have gained prominence in the machine learning literature (Zhang et al., 2017; Pearl, 2018).

The new objective is to train a model while incorporating constraints of the form $\mathrm{Pr}\left({\text{X}}_{-A}|do\left(A=\alpha \right)\right)=\mathrm{Pr}\left({\text{X}}_{-A}|do\left(A=\alpha^{\prime }\right)\right)$, where **X**_−*A*_ denotes the set of all the model's variables, excluding *A*. Constraints of this kind have powerful implications regarding the causal mechanisms between *A* and the rest of the variables. This could be seen clearly when considering similar constraints to the one above, such as Pr(**X**_−*A*_|*do*(*A* = α)) = Pr(**X**_−*A*_), which means that setting *A* to a certain value does not influence the distribution of the rest of the variables. Intuitively, this means that *A* has no causal influence on any of the remaining variables.

As we have mentioned in a previous section, we will base our approach on a well-known formula connecting the interventional to the observational distribution (Pearl, 2009b):

$$\begin{array}{l} \mathrm{Pr}\left(X_{-A}|do\left(A=\alpha \right)\right)=\frac{\mathrm{Pr}\left(X_{-A},A=\alpha \right)}{\mathrm{Pr}\left(A=\alpha |pa_{A}\right)} \end{array}$$

Depending on the application, it is possible there is enough background knowledge available to specify *pa*_*A*_. There might be other applications though, where this is not an option, due to the complexity of the problem or insufficient a priori information. In these cases, methods from the field of *feature selection* (Guyon and Elisseeff, 2003) could be utilized. The aim of these methods is to identify the Markov Blanket of a set of variables, so it is closely related to specifying the parents of a variable. Conditioning on the Markov Blanket, instead of just the parents, can serve as an approximation of the desired distribution, so there is a wide range of methods (Zhang et al., 2011; Peters et al., 2016; Zheng et al., 2018) for performing this step. Assuming we possess the parents of the variable of interest, we can show the following:

**Theorem 2**. *Let* S *be an SPN representing the joint distribution of variables X*_1_, ⋯ , *X*_*n*_. *Let X*_*i*_ *be a binary variable, then the constraint* Pr(***X***_−*i*_|*do*(*X*_*i*_ = 0)) = Pr(***X***_−*i*_|*do*(*X*_*i*_ = 1)) *is equivalent to a multivariate linear system of equations on the SPN's parameters*.

*Proof*. We will prove this, following the same reasoning as in the previous proof, so we first need to rewrite the given constraint:

$$\begin{array}{l} \mathrm{Pr}\left(X_{-i}|do\left(X_{i}=0\right)\right)=\mathrm{Pr}\left(X_{-i}|do\left(X_{i}=1\right)\right)\\ \Rightarrow \frac{\mathrm{Pr}\left(X_{-i},X_{i}=0\right)}{\mathrm{Pr}\left(X_{i}=0|pa_{X_{i}}\right)}=\frac{\mathrm{Pr}\left(X_{-i},X_{i}=1\right)}{\mathrm{Pr}\left(X_{i}=1|pa_{X_{i}}\right)}\\ \Rightarrow \mathrm{Pr}\left(X_{-i},X_{i}=0\right)\cdot \mathrm{Pr}\left(X_{i}=1|pa_{X_{i}}\right)\\ =\mathrm{Pr}\left(X_{-i},X_{i}=1\right)\cdot \mathrm{Pr}\left(X_{i}=0|pa_{X_{i}}\right)\\ \Rightarrow \mathrm{Pr}\left(X_{-i},X_{i}=0\right)\cdot \mathrm{Pr}\left(X_{i}=1,pa_{X_{i}}\right)\\ =\mathrm{Pr}\left(X_{-i},X_{i}=1\right)\cdot \mathrm{Pr}\left(X_{i}=0,pa_{X_{i}}\right) \end{array}$$

The next step is to express these probabilities in terms of the network polynomial and substitute them to the above expression. Since these computations are lengthy and routine, we will not present them here. The important observation is that it is not difficult to see that we end up with a system of multivariate polynomials, in this case, too. To prove they are linear ones as well, it suffices to note that in both products Pr(**X**_−*i*_, *X*_*i*_ = 0) · Pr(*X*_*i*_ = 1, *pa*_*X*_*i*__) and Pr(**X**_−*i*_, *X*_*i*_ = 1) · Pr(*X*_*i*_ = 0, *pa*_*X*_*i*__), the set of parameters involved in the first factor is disjoint with the one appearing in the second factor, since the parameters that remain after setting *X*_*i*_ = 0 vanish when setting *X*_*i*_ = 1 (and vice versa).

Following the discussion about extending the binary case to the general case, after Theorem 1, it should not be surprising that the same holds true for Theorem 2 as well. The main observation is the same, that enforcing an interventional constraint for the non-binary case can be transformed into a system of equations which can be addressed using Theorem 2, resulting in a system of multivariate linear equations. For example, the same argument that was presented in the previous section, ensures that if *X*_*i*_ ∈ {0, 1, ⋯ , *k* − 1} then the number of equations is scaled by a factor of *k* − 1.

### 4.3. Independence Constraints

The last kind of constraints we will present are those enforcing independence between variables. There are some already existing approaches, such as Xu et al. (2018), allowing for incorporating rules expressed as propositional formulas within the model, in order for example to impose certain structure to the outcome variable, but doing the same with probabilistic ones still poses a major challenge.

Using reasoning analogous to the previous cases, it is possible to incorporate conditional independence or context specific information within the model. Although similar in spirit, since usually both of them relies on conditioning, each one provides different insights about the problem at hand. So, for example, conditional constraints could be of the form: if we know the value of a variable, *Z*, then *A* and *B* are independent. On the other hand, context specific independence is stronger, since it might state that only when *Z* = *z* we know that *A* and *B* are independent. However, it is not difficult to see that each of these independencies can be expressed as Pr(*A, B*|*Z*) = Pr(*A*|*Z*) Pr(*B*|*Z*) and Pr(*A, B*|*Z* = *z*) = Pr(*A*|*Z* = *z*) Pr(*B*|*Z* = *z*), respectively.

Assuming, as before, that the objective is to train an SPN satisfying constraints like the above, we can show that it amounts to optimizing a function over a set of multivariate quadratic polynomial constraints.

**Theorem 3**. *Let* S *be an SPN representing the joint distribution of variables X*_1_, ⋯ , *X*_*n*_. *Let X*_*i*_, *X*_*j*_ *be two binary variables, then the constraint* Pr(*X*_*i*_, *X*_*j*_) = Pr(*X*_*i*_) · Pr(*X*_*j*_) *is equivalent to a multivariate quadratic system of four equations on the SPN's parameters*.

*Proof*. To prove this result it is not necessary to rewrite the given constraint, so we can start with expressing these probabilities in terms of S:

$$\begin{array}{l} \mathrm{Pr}\left(X_{i},X_{j}\right)=\underset{x:x_{i},x_{j}}{\sum }f\left(x\right){𝟙}_{x_{i}}{𝟙}_{x_{j}}+\underset{x:\neg x_{i},x_{j}}{\sum }f\left(x\right){𝟙}_{\neg x_{i}}{𝟙}_{x_{j}}\\ \;+\underset{x:x_{i},\neg x_{j}}{\sum }f\left(x\right){𝟙}_{x_{i}}{𝟙}_{\neg x_{j}}+\underset{x:\neg x_{i},\neg x_{j}}{\sum }f\left(x\right){𝟙}_{\neg x_{i}}{𝟙}_{\neg x_{j}}\\ \mathrm{Pr}\left(X_{i}\right)=\underset{x:x_{i}}{\sum }f\left(x\right){𝟙}_{x_{i}}+\underset{x:\neg x_{i}}{\sum }f\left(x\right){𝟙}_{\neg x_{i}}\\ \mathrm{Pr}\left(X_{j}\right)=\underset{x:x_{j}}{\sum }f\left(x\right){𝟙}_{x_{j}}+\underset{x:\neg x_{j}}{\sum }f\left(x\right){𝟙}_{\neg x_{j}} \end{array}$$

Next, we substitute these quantities to the constraint's equation, so we get that:

$$\begin{array}{l} \underset{x:x_{i},x_{j}}{\sum }f\left(x\right){𝟙}_{x_{i}}{𝟙}_{x_{j}}+\underset{x:\neg x_{i},x_{j}}{\sum }f\left(x\right){𝟙}_{\neg x_{i}}{𝟙}_{x_{j}}+\underset{x:x_{i},\neg x_{j}}{\sum }f\left(x\right){𝟙}_{x_{i}}{𝟙}_{\neg x_{j}}\\ +\underset{x:\neg x_{i},\neg x_{j}}{\sum }f\left(x\right){𝟙}_{\neg x_{i}}{𝟙}_{\neg x_{j}}=\underset{x:x_{i}}{\sum }f\left(x\right)\cdot \underset{x:x_{j}}{\sum }f\left(x\right){𝟙}_{x_{i}}{𝟙}_{x_{j}}\\ +\underset{x:x_{i}}{\sum }f\left(x\right)\cdot \underset{x:\neg x_{j}}{\sum }f\left(x\right){𝟙}_{x_{i}}{𝟙}_{\neg x_{j}}+\underset{x:\neg x_{i}}{\sum }f\left(x\right)\cdot \underset{x:x_{j}}{\sum }f\left(x\right){𝟙}_{\neg x_{i}}{𝟙}_{x_{j}}\\ +\underset{x:\neg x_{i}}{\sum }f\left(x\right)\cdot \underset{x:\neg x_{j}}{\sum }f\left(x\right){𝟙}_{\neg x_{i}}{𝟙}_{\neg x_{j}} \end{array}$$

Equating the coefficients we get the following system of equations:

$$\begin{array}{l} \underset{x:x_{i},x_{j}}{\sum }f\left(x\right)=\underset{x:x_{i}}{\sum }f\left(x\right)\cdot \underset{x:x_{j}}{\sum }f\left(x\right)\\ \underset{x:\neg x_{i},x_{j}}{\sum }f\left(x\right)=\underset{x:\neg x_{i}}{\sum }f\left(x\right)\cdot \underset{x:x_{j}}{\sum }f\left(x\right)\\ \underset{x:x_{i},\neg x_{j}}{\sum }f\left(x\right)=\underset{x:x_{i}}{\sum }f\left(x\right)\cdot \underset{x:\neg x_{j}}{\sum }f\left(x\right)\\ \underset{x:\neg x_{i},\neg x_{j}}{\sum }f\left(x\right)=\underset{x:\neg x_{i}}{\sum }f\left(x\right)\cdot \underset{x:\neg x_{j}}{\sum }f\left(x\right) \end{array}$$

Each of these equations correspond to a multivariate polynomial, as in all the previous cases, but this time they are quadratic, instead. This is because, in each equation, the sums appearing on the right hand side have some terms in common. For example, looking at the first equation, the assignment setting all the variables equal to 1 is compatible with both summations, so the term *f*(*x*_1_, ⋯ , *x*_*n*_) appears in both of them. Clearly, by multiplying them we end up with a squared parameter.

Concluding this section, we should note that Theorem 3 can be extended to the general case, too. Following the same arguments, and assuming that *X*_*i*_ ∈ {0, 1, ⋯ , *m* − 1}, *X*_*j*_ ∈ {0, 1, ⋯ , *k* − 1}, then enforcing an independence constraint results in a multivariate quadratic system of *mk* equations.

## 5. Applying the Framework

In this section we will demonstrate how to derive the system of equations that correspond to a single constraint. Let's assume we would like to train an SPN, S, over three binary variables, *X*_1_, *X*_2_, *X*_3_, satisfying the property that *X*_1_ and *X*_2_ are independent. The canonical polynomial of S (Darwiche, 2003) is:

(2) $$\begin{array}{l} S\left(X_{1},X_{2},X_{3},\neg X_{1},\neg X_{2},\neg X_{3}\right)=\theta_{1}X_{1}X_{2}X_{3}+\theta_{2}\neg X_{1}X_{2}X_{3}\\ +\theta_{3}X_{1}\neg X_{2}X_{3}+\theta_{4}\neg X_{1}\neg X_{2}X_{3}+\theta_{5}X_{1}\neg X_{2}\neg X_{3}+\theta_{6}\neg X_{1}X_{2}\neg X_{3}\\ +\theta_{7}X_{1}X_{2}\neg X_{3}+\theta_{8}\neg X_{1}\neg X_{2}\neg X_{3} \end{array}$$

where each θ_*i*_ is equal to the probability of the specific configuration of *X*_1_, *X*_2_, *X*_3_ following it, so, for example, in the term θ_5_*X*_1_¬*X*_2_¬*X*_3_, θ_5_ = Pr(*X*_1_, ¬*X*_2_, ¬*X*_3_)

**Algorithm 1:** Training with soft constraints

**Input**: SPN structure S, dataset *D*, constraints *C*_*n*_, hyperparameters α_*n*_, learning rate γ
**Output**: An SPN with parameters **w**
1 Initialize **w**;
2 **repeat**;
3 Sample a mini batch *M*, from *D*;
4 **for all** *m* ∈ *M* **do**;
5 **w**^(*k*)^ ← **w**^(*k*−1)^ + γ(∇_**w**_S(m) + $\underset{n}{\sum }\alpha_{n}\nabla_{\text{w}}C_{n})$;
6 **end for**;
7 **until convergence**
8 S ← *NormalizeWeights(*S*)*;
9 **return** S

The joint probability of, say, *X*_1_, *X*_2_ is given by the above, after substituting both *X*_3_, ¬*X*_3_ by 1, so Pr(*X*_1_, *X*_2_) = S(*X*_1_, *X*_2_, 1, ¬*X*_1_, ¬*X*_2_, 1). In the same way, Pr(*X*_1_) = S(*X*_1_, 1, 1, ¬*X*_1_, 1, 1) and Pr(*X*_2_) = S(1, *X*_2_, 1, 1, ¬*X*_2_, 1).

At this point, it is time to utilize the condition we would like to enforce, Pr(*X*_1_, *X*_2_) = Pr(*X*_1_)Pr(*X*_2_). Substituting these probabilities by the corresponding polynomial, yields the following:

$$\begin{array}{l} \left(\theta_{1}+\theta_{7}\right)X_{1}X_{2}+\left(\theta_{3}+\theta_{5}\right)X_{1}\neg X_{2}+\left(\theta_{2}+\theta_{6}\right)\neg X_{1}X_{2}\\ +\left(\theta_{4}+\theta_{8}\right)\neg X_{1}\neg X_{2}=\left(\theta_{1}+\theta_{3}+\theta_{5}+\theta_{7}\right)\cdot (\theta_{1}+\theta_{2}\\ +\theta_{6}+\theta_{7})X_{1}X_{2}+\left(\theta_{1}+\theta_{3}+\theta_{5}+\theta_{7}\right)\cdot \left(\theta_{3}+\theta_{4}+\theta_{5}+\theta_{8}\right)\\ X_{1}\neg X_{2}+\left(\theta_{2}+\theta_{4}+\theta_{6}+\theta_{8}\right)\cdot \left(\theta_{1}+\theta_{2}+\theta_{6}+\theta_{7}\right)\\ \neg X_{1}X_{2}+\left(\theta_{2}+\theta_{4}+\theta_{6}+\theta_{8}\right)\cdot \left(\theta_{3}+\theta_{4}+\theta_{5}+\theta_{8}\right)\neg X_{1}\neg X_{2} \end{array}$$

This is an equivalence between polynomials, so all the coefficients must be equal, meaning that:

$$\begin{array}{l} \theta_{1}+\theta_{7}=\left(\theta_{1}+\theta_{3}+\theta_{5}+\theta_{7}\right)\cdot \left(\theta_{1}+\theta_{2}+\theta_{6}+\theta_{7}\right),\\ \theta_{3}+\theta_{5}=\left(\theta_{1}+\theta_{3}+\theta_{5}+\theta_{7}\right)\cdot \left(\theta_{3}+\theta_{4}+\theta_{5}+\theta_{8}\right)\\ \theta_{2}+\theta_{6}=\left(\theta_{2}+\theta_{4}+\theta_{6}+\theta_{8}\right)\cdot \left(\theta_{1}+\theta_{2}+\theta_{6}+\theta_{7}\right),\;\\ \theta_{4}+\theta_{8}=\left(\theta_{2}+\theta_{4}+\theta_{6}+\theta_{8}\right)\cdot \left(\theta_{3}+\theta_{4}+\theta_{5}+\theta_{8}\right) \end{array}$$

Each θ_*i*_ in the resulting equations has probabilistic semantics, so we could perform a sanity check, by rewriting the system in terms of these probabilities. This will provide some insights on the underlying constraints, as well as some hints on alternative ways to incorporate the constraints in the model.

$$\begin{array}{l} \theta_{1}+\theta_{7}=\mathrm{Pr}\left(X_{1},X_{2}\right),\theta_{2}+\theta_{6}=\mathrm{Pr}\left(\neg X_{1},X_{2}\right),\\ \theta_{3}+\theta_{5}=\mathrm{Pr}\left(X_{1},\neg X_{2}\right),\theta_{4}+\theta_{8}=\mathrm{Pr}\left(\neg X_{1},\neg X_{2}\right)\\ \theta_{1}+\theta_{3}+\theta_{5}+\theta_{7}=\mathrm{Pr}\left(X_{1}\right),\theta_{1}+\theta_{2}+\theta_{6}+\theta_{7}=\mathrm{Pr}\left(X_{2}\right),\\ \theta_{3}+\theta_{4}+\theta_{5}+\theta_{8}=\mathrm{Pr}\left(\neg X_{2}\right),\theta_{2}+\theta_{4}+\theta_{6}+\theta_{8}=\mathrm{Pr}\left(\neg X_{1}\right) \end{array}$$

Substituting all these quantities to the original system, we get the following constraints:

(3) $$\begin{array}{l} \mathrm{Pr}\left(X_{1},X_{2}\right)=\mathrm{Pr}\left(X_{1}\right)\cdot \mathrm{Pr}\left(X_{2}\right),\;\mathrm{Pr}\left(X_{1},\neg X_{2}\right)=\mathrm{Pr}\left(X_{1}\right)\cdot \mathrm{Pr}\left(\neg X_{2}\right),\\ \mathrm{Pr}\left(\neg X_{1},X_{2}\right)=\mathrm{Pr}\left(\neg X_{1}\right)\cdot \mathrm{Pr}\left(X_{2}\right),\;\mathrm{Pr}\left(\neg X_{1},\neg X_{2}\right)\\ =\mathrm{Pr}\left(\neg X_{1}\right)\cdot \mathrm{Pr}\left(\neg X_{2}\right) \end{array}$$

At this point, it might appear like the proposed framework can only be applied when having a non-factorized, canonical, polynomial representation, such as *S*. However, a closer inspection of Equation (3), hints at a way to treat the factorized case as well. The main insight is that the only essential requirement is to be able to compute all the probabilities in (3). Furthermore, SPNs can compute these probabilities regardless of whether they encode the canonical polynomial or one of its factorized representations. Of course, the more compact a SPN is, the more efficient is inference utilizing it, but it is always possible to infer these probabilities. After obtaining all the necessary quantities (each one requiring a single pass over the SPN), we can substitute them into the equations in (3). This process leads to the final system of equations, which is expressed entirely in terms of the SPN's parameters.

The same reasoning can be extended to all the considered constraints, not only the independence ones. Taking a look at the proofs, we see that all of them share a central argument; that is, substituting the probabilistic quantities appearing in a constraint with the corresponding SPN outcomes, thus obtaining a system involving only the SPN's parameters. As discussed in the preceding paragraph, all the necessary probabilities can be inferred, when using a SPN. For example, enforcing a conditional constraint of the form Pr(*X*_1_|*X*_2_) = Pr(*X*_1_|¬*X*_2_), requires computing Pr(*X*_1_, *X*_2_), Pr(*X*_1_, ¬*X*_2_), Pr(*X*_2_), Pr(¬*X*_2_) (using a SPN) and substituting them into the equation Pr(*X*_1_, *X*_2_) · Pr(¬*X*_2_) = Pr(*X*_1_, ¬*X*_2_)·Pr(*X*_2_). The final equation involves only the SPN parameters, so we are now ready to proceed to the optimization step.

## 6. Model Training

The previous sections introduced the connection between the network polynomial of an SPN and various probabilistic queries. In this section we are going to discuss how to utilize these insights in order to train SPNs that incorporate probabilistic constraints. Many recent approaches (Gens and Domingos, 2013; Rooshenas and Lowd, 2014; Adel et al., 2015) attempt to learn both the structure and the parameters of a SPN. The tuning of the parameters' values is usually achieved using a heuristic, such as the proportion of the training instances in a sum node. However, as noted in Zhao et al. (2016), first learning the structure, using some of the aforementioned approaches, and then fitting the parameters, yields better results. In our presentation we are going to follow the latter approach, since our focus is on learning the model's parameters. In what follows we are going to assume that the SPN structure is known, obtained using some of the existing algorithms, such as Gens and Domingos (2013).

Incorporating soft constraints is equivalent to adding new terms in the objective function. In our case, all of these terms are differentiable, since they are polynomials, so any standard optimization algorithm could be utilized to train the model. Algorithm 1 describes a pipeline for carrying out this procedure. Apart from including the extra terms in the objective function, we also allow for a hyperparameter, α, so it is possible to adjust the relative importance of each constraint. Furthermore, we would like to note that it is not necessary to explicitly compute the constraints in terms of the parameters, since the algorithm only utilizes their gradient. In turn, the fact that each constraint involves probabilistic quantities, which correspond to sub-SPNs, leads to the observation that all derivatives can be readily computed by combining the chain rule and the remarks about SPN differentiation in Darwiche (2003) and Poon and Domingos (2011). The result of the optimization routine is an unormalized SPN, so the last step in Algorithm 1 is to normalize it, as described in Peharz et al. (2015).

In contrast, if they are treated as hard constraints, projected gradient descent or approaches like the one developed in Marquez Neila et al. (2017) would need to be used to train the SPN. Algorithm 2 is a variation of Algorithm 1, adapted to train an SPN under hard constraints. The modification lies on the fact that after the weights are updated, then they are projected on the space defined by the constraints, using the **P**_*C*_1_, …, *C*_*n*__(·) operator, see Zhao et al. (2016) and Marquez Neila et al. (2017) for different projection techniques and their effect on the resulting solutions. For this variant our results are essential, since the equations cannot be handled implicitly, as was possible with soft constraints. Our approach, as seen in this example, provides a way to recover exactly these equations, so training with hard constraints can be made possible. Furthermore, although the discussion has focused on the binary case, the same holds true in the general case as well. The only adjustment needed would be to incorporate more equations into the system. However, we should note that while the added equations would, of course, lead to a larger system for the optimization routine to solve, the scaling factor is only moderate, as discussed in section 4. Furthermore, the degree of the resulting equations is not affected by extending the results to the general case, meaning it is quadratic, at most. In our opinion, although incorporating hard constraints is more involving, it is worth exploring this approach, since using soft constraints, as in Xu et al. (2018), does not guarantee the resulting model will satisfy them.

**Algorithm 2:** Training with hard constraints

**Input**: SPN structure S, dataset *D*, constraints *C*_*n*_, hyperparameters α_*n*_, learning rate γ
**Output**: An SPN with parameters **w**
1 Initialize **w**;
2 **repeat**;
3 Sample a mini batch *M*, from *D*;
4 **for all** *m* ∈ *M* **do**;
5 **w**^(*k*)^ ← ${\text{P}}_{C_{1},\ldots ,C_{n}}({\text{w}}^{\left(k-1\right)}$ + γ∇_**w**_S(m));
6 **end for**;
7 **until convergence**
8 S ← *NormalizeWeights(*S*)*;
9 **return** S

## 7. Conclusions

In the previous sections we presented an approach allowing to train SPNs under probabilistic constraints. SPNs are tractable models, meaning that probabilistic inference is efficient, since marginal or conditional queries can be computed in time linear in its size. This is an appealing property, because otherwise additional steps, such as MCMC sampling, would be necessary in order to perform inference. Taking that into account, SPNs can not only incorporate probabilistic assumptions, but they can also easily compute such queries in polynomial time.

An other interesting point is that our work could be seen as related to the work that has been done in the field of *Fairness in AI*, but from a generative modeling point of view. The main objective in the field is to formalize criteria leading to fair predictions, and train models satisfying these criteria. For example, enforcing a condition, such as Pr(ŷ = 1|*a* = 0) = Pr(ŷ = 1|*a* = 1), where *a* is a protected binary attribute and ŷ is the model's prediction, has been proposed (Zemel et al., 2013). In our setting there is no predicted variable, so this condition cannot be applied. However, an analogous condition could be utilized when dealing with generative modeling, such as Pr(*y* = 1|*a* = 0) = Pr(*y* = 1|*a* = 1).

In this work we provided a way to equip SPNs with background information. This adds to the growing literature on constraints and machine learning that is emerging recently. The key difference in our results is that it is proven for generative models, unlike the majority of the existing work, as well as it exhibits how the model's intrinsic architecture can be utilized to do so, allowing us to recover a system of equations. We hope the results of this paper will lead to a new range of applications making use of tractable generative models that allow the incorporation of non-trivial probabilistic prior knowledge.

There is a number of promising directions regarding future research. In our presentation we only consider probabilistic constraints, so extending our results to account for propositional background knowledge is an immediate next step. Furthermore, we only consider discrete SPNs in this work, but it may be possible to extend these results to continuous SPNs, perhaps utilizing distribution selectors, as in Peharz et al. (2015). Exploring ways to incorporate inequality constraints as well, makes for another interesting open question. One of our future endeavors is to utilize the Convex Concave Procedure interpretation of the SPN parameter learning, given in Zhao et al. (2016), and combine it with prior work in the field dealing with inequality constraints, such as Lipp and Boyd (2016).

## Data Availability Statement

The original contributions presented in the study are included in the article/supplementary material, further inquiries can be directed to the corresponding author/s.

## Author Contributions

IP and VB conceived of the presented idea. IP developed the theory and the proofs of the theorems. VB supervised the writing of the manuscript. Both authors contributed to the final version of the manuscript.

## Conflict of Interest

The authors declare that the research was conducted in the absence of any commercial or financial relationships that could be construed as a potential conflict of interest.
